# Diagnostic Significance of White Blood Cell Count and C-Reactive Protein in Neonatal Sepsis; Asella Referral Hospital, South East Ethiopia

**DOI:** 10.2174/1874285801812010209

**Published:** 2018-06-29

**Authors:** Abebe Sorsa

**Affiliations:** Asella College of Health Science, Asella, Ethiopia

**Keywords:** Neonatal sepsis, WBC count, CRP, Sensitivity, Specificity, PPV, NPV

## Abstract

**Introduction:**

Nowadays various biochemical markers, such as C-Reactive Protein (CRP), Procalcitonin and tumor necrosis factor alpha, have been proposed as a potential marker for screening neonatal sepsis. In the current study, we tried to see the diagnostic significance of White Blood Cell (WBC) count and CRP in diagnostic screening of neonatal sepsis.

**Methods:**

A prospective cross-sectional study was conducted from May 2016 to April 2017 in Asella Teaching and Referral Hospital. Data were entered into EPI-INFO version 3.5.1 for cleanup and then exported to SPSS version 17 for further analysis. Sensitivity, specificity, positive predictive value (PPV) and negative predictive values (NPV) were used to assess the accuracy of CRP and WBC count taking blood culture as gold standard.

**Results:**

Data of 303 neonates with clinical sepsis were analyzed. Positive CRP and abnormal WBC were reported in 136(45%) and 99(32.7%) of study subjects respectively. Blood culture turned to be positive in 88(29.4%) of study subjects. The Sensitivity, Specificity, PPV and NPV of WBC count were 59.5 %, 79.6%, 52%, 64.5% respectively while the sensitivity, specificity, PPV and NPV of CRP were 65.6%, 78%, 42% and 91% respectively. By combining both WBC and CRP, the sensitivity, specificity, PPV and NPV improve to 78.5%, 83%, 60% and 93% respectively. CRP positivity rate was comparable across gram positive and gram negative bacteria while high WBC count were more reported among gram positive sepsis than gram negative ( OR 4.8, (95% CI 1.45-15.87, P 0.01)

**Conclusion:**

Based on this study’s finding, it can be concluded that CRP alone or in combination with WBC count showed better diagnostic accuracy in neonatal sepsis.

## 1 INTRODUCTION

Early recognition of sepsis in the neonate is one of the most difficult problems facing clinicians today [1, 2, 10]. Delayed diagnosis and inappropriate treatment of neonatal sepsis has been associated with an increased systemic complication with high mortality rate. The currently accessible tests are not able to provide complete diagnostic accuracy with an accepted margin of false-negative as well as false-positive results; the utility of auxiliary laboratory

test therefore primarily depends on the clinical condition of the neonate [3]. On the other hand; even though blood culture is said to be the gold standard diagnostic test for sepsis, there are a number of limitations including; unavailability in the majority of developing country, associated technical problem and it takes more than three days to see at least the first preliminary result [4, 6]. As a result, the diagnosis of neonatal sepsis is based on clinical assessment and the management also rely on empirical treatment protocol which usually results in unnecessary hospital stay, increase irrational use of antibiotics and incur an unnecessary cost for the family [4, 5]. However; nowadays various biochemical markers, such as C-Reactive Protein (CRP), Procalcitonin (PCT), and tumor necrosis factor alpha (TNF-α), have been proposed as a potential marker for diagnosis of neonatal infection [6]. CRP is a pentraxin protein which plays an important role in inflammatory and/or infectious stimuli, thus being regarded as acute phase reactant in neonatal sepsis. CRP is one of the most studied bio markers of neonatal sepsis which is available at most laboratory centers. Diagnostic accuracy clearly improves by performing serial determinations and by the combination with other biomarkers such as interleukins or procalcitonin. Most previous studies have established that CRP is a useful diagnostic test for the early stages of neonatal sepsis reaching a peak during the first 24-48 hours with better sensitivity and specificity. Its serum half time is 5 to 7 hours [5-8]. There are several methods available for measuring CRP. Some are fully automated and quantitative, whereas others are semiquantitative or qualitative and manual [4-7].

On the other hand, white blood cell count (WBC) is one of the routinely done diagnostic tests for sepsis work up and many aspects of the leukocyte count have been examined for their predictive value in diagnosing sepsis. Leukocytosis or leukopenia, defined as WBC count of greater than 20,000/mm^3^ or less than 5000/mm^3^, respectively, was believed to be reliable indicators of infection but now are known to be insensitive and nonspecific [4, 5, 8]. Furthermore, a single leukocyte count obtained shortly after birth is not adequately sensitive for diagnosing neonatal sepsis. A multicenter study in which both blood culture and WBC were performed after 24 hours of life, low WBC and neutrophils associated with higher risk of culture-proven sepsis [8-11]. There are few studies which evaluated the significance of CRP and WBC count in the diagnosis of neonatal sepsis in developing countries, and as to author’s knowledge, there is no study from Ethiopia. Our current study is unique in that it mainly focused on neonatal sepsis diagnostic approach which has a potential to change the current recommendation.

Therefore, the objective of this study was to evaluate the significance of CRP and WBC count in the diagnosis of neonatal sepsis.

## 2 METHODS AND MATERIALS

### 2.1 Study Design

Hospital-based prospective study was conducted from April 2016 to May 2017.

### 2.2 Study Setting (Area)

The study was conducted at Asella Teaching and Referral Hospital (ATRH) which is one of the Federal referral hospitals located in Arsi Zone, Oromia region, South East Ethiopia.

The NICU is one of the busiest services where the total number of admissions per year is close to 1200 neonates.

### 2.3 Source Population

All neonates admitted to NICU at ATRH from May 2016 to April 2017.

### 2.4 Study Population

 All neonates who fulfilled inclusion criteria.

### 2.5 Inclusion Criteria

Neonates with the clinical diagnosis of sepsis based on the presence of two of the following risk factors and/or clinical features of bacterial infections based on WHO-AIIMS 2014 protocol.

Low birth weight (<2500 grams) or prematurity(<37 weeks of gestation).

Presence maternal febrile illness within 2 weeks prior to delivery.

Foul smelling and/or meconium stained amniotic liquid.

Suspected chorioamnionitis.

Rupture of membranes >18 hours.

Prolonged labor (the sum of 1^st^ and 2^nd^ stage of labor) >24 hrs.

Perinatal asphyxia (Apgar score <4 at 1 minute).

Clinical signs of sepsis (poor reflexes, lethargy, respiratory distress, bradycardia, apnea, fever, convulsions, abdominal distension, and bleeding.

### 2.6 Exclusion Criteria

Neonate whose mother available was not to give consent and interview.

### 2.7 Operational Definition

Early Onset Neonatal Sepsis (EONS): sepsis diagnosed in the first six days of life [12].

Late Onset Neonatal Sepsis (LONS): sepsis diagnosed between the age of 7 to 28 days of life [12].

Premature Rupture of Membrane (PROM): rupture of membrane before the onset of life [12].

**Prolonged PROM:** PROM lasting for more than 18 hrs [12].

**Prolonged labor:** Total duration of labor lasting for more than 24 hrs [12].

Low APGAR score is the score <6 [11].

Sample size determination and sampling technique.

The sample size was estimated using single proportion formula and taking the prevalence of neonatal sepsis to be 30% [11, 13] and by anticipating sensitivity and specificity of 95% by allowing 5% margin of error a minimum sample size obtained was 303 neonates.

### 2.8 Data Collection Method

#### 2.8.1 Clinical Data

Using WHO-AIIMS 2014 protocol for clinical diagnosis of neonatal sepsis and by reviewing other literature, a structured questionnaire tool was prepared. Important demographic variables, risk factors for neonatal sepsis and clinical feature of neonatal sepsis were capture to the tool.

#### 2.8.2 Laboratory Data

**White Blood Culture (WBC):** WBC count of >20,000 is defined as leukocytocis and count less than 5,000 defined as leucopenia [4, 5].

**C-Reactive Protein (CRP)**: C-Reactive Protein was determined after 24 hours of postnatal age using qualitative test. The latex reagent was stored at a temperature of 2-8°C. The test is based on agglutination principle. The latex reagent was shaken gently and one drop of it was mixed with a drop of serum on the slide and mixed with a mixer. The slide was rocked forth and back for two minutes for observing macroagglutination. CRP starts to rise after six hours of onset of the inflammatory process and reach a peak after 24 hours. For this reason, CRP was determined after 24 hours of life.

**Blood Culture:** Blood sample was taken using aseptic technique for culture.

## 2.9 Data Analysis

Data were entered into EPI-INFO version 3.5.1 for cleanup; and then were exported to SPSS version 17 for further analysis. Sensitivity, Specificity, Positive Predictive Value (PPV) and Negative Predictive Value (NPV) were used to assess the validity, reliability of single determination of CRP and WBC taking Blood culture as gold standard. Frequencies, proportion and binary logistic regressions and odds ratios with 95% confidence intervals were used to assess factors associated with abnormal WBC or CRP. P-values < 0.05 was considered statistically significant.

## 2.10 Ethical Consideration

The research proposal was presented and approved by Institutional Review Board of Arsi University and ethical clearance was obtained. Individual verbal consent was obtained from mothers/caregivers of eligible neonate before starting interviews/Laboratory sampling. The participation was purely voluntary. Confidentiality and privacy was maintained by conducting interviews in separate space as much as possible. Benefits of the laboratory test were clearly communicated to mothers/caregivers and patients whose blood culture results showed positive growth, susceptibility based antibiotics were started.

## 3 RESULTS

From 322 study subjects, eleven of them were excluded based on exclusion criteria, eight laboratory results were incomplete or missing. As a result, data’s of 303 subjects with complete information was subjected for analysis. Male contributed for 198 (65.3%) and female were 105 (34.7%) resulting in male to female ratio of 1.88: 1. Of the total study subjects with clinical neonatal sepsis, 64 (21.1%) were born preterm, 256 (84.6%) were born at health facilities (hospitals/health center) and 47 (15.4%) were born at home. Regarding the mode of delivery; 222 (73.1%) were delivered by spontaneous vaginal delivery (SVD) whereas 73 (24.1%) were delivered by cesarean section (Table **1**). Of newborns whose birth weight was registered, 56 (18.4%) neonates were having low birth weight (<2500 g) out of which 13 (22.45%) had very low birth weight (<1500 g). Respiratory distress was the most common clinical presentation of neonatal sepsis, which accounted for about 192 (63.3%).

From the total cases of clinical neonatal sepsis, 185 (61.2%) were recognized as EONS and 118 (38.8%) were recognized as LONS according to infants’ age at the time of presentation. Neonates born to mothers with inadequate ANC visits and fever were having a higher risk of clinical sepsis; AOR 2.088 (95% CI 1.013-4.302) and 2.214 (95% CI 0.753-6.509).

### 3.1 Laboratory Results

#### 3.1.1 C-Reactive Protein

All 303 neonates with the diagnosis of clinical sepsis, underwent CRP determination where positive results were reported in 136 (45%) of cases.

### 3.2 White Blood Cell Results

WBC was determined for all study subjects. Abnormalities in the WBC count were found in 99 (32.7%) neonates with 9 (2.9%) having leucopenia (WBC < 5,000/mm3), 90 (29.8%) leukocytosis (WBC > 20,000/mm3).

### 3.3 Blood Culture Results

A total of 88 (29.3%) of positive blood culture was reported and when to disaggregate to the age of presentation; 37 (42.0%) culture positive results were from early-onset sepsis and 51 (59.0%) were from late-onset sepsis. Patients with leukocytosis have a higher risk of culture-proven sepsis, AOR of 5.68, 95%CI 3.415-9.013, p<0.001. Similarly, neonates with positive CRP have a higher risk of culture-confirmed sepsis, AOR of 5.71, 95%CI 3.26-10.00, p<0.001)

### 3.4 Sensitivity, Specificity, PPV and NPV of CRP and WBC

**Fig. (1):**
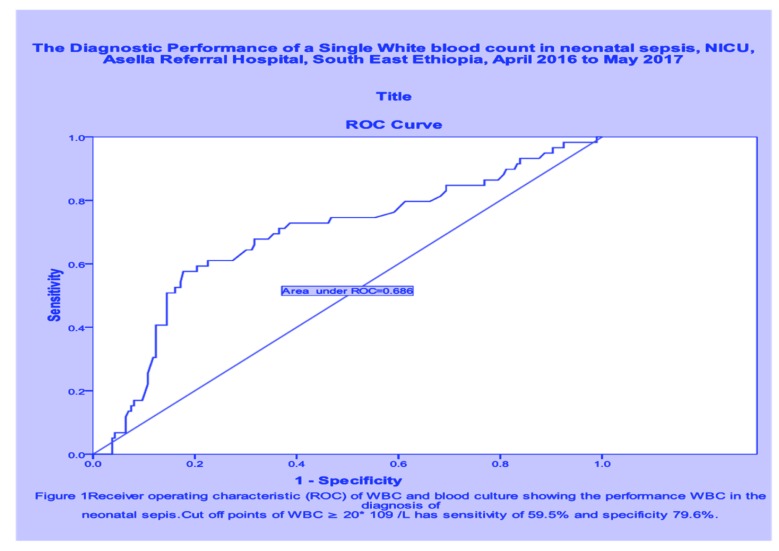
Receiver operating characteristics (ROC) of WBC and blood culture showing the performance WBC in the neonatal sepis. Cut off points of WBC ≥ 20* 109/ L has sensitivity of 59.5% and specificity 79.6%.

The Sensitivity and Specificity of CRP were 65.6% and 78% with positive predictive value (PPV) and negative predictive value (NPV) of 42% and 91%, respectively. On the other hand; the Sensitivity and Specificity of WBC were 59.5% and 79.6% with PPV and NPV of 52% and 64.5% respectively, and area under the curve of 0.686 (Fig. **1**). When CRP is combined with WBC count the sensitivity and specificity became much better (Table **2**). There is no significant difference with CRP positivity across both gram positive and gram negative bacteria (Table **2**). There is a higher rate of leukocytosis among gram-positive sepsis than gram-negative sepsis (OR 4.8, (95% CI 1.45-15.87, P 0.01). CRP positivity rate and leukocytosis don’t differ significantly between both early and late onset sepsis.

### 3.5 Factors Associated With Positive CRP and Neonatal Sepsis

On bivariate analysis first and fifth APGAR score, Gestational age of the newborn, birth weight of the newborn, meconium staine liquor, the temperature at admission, WBC count at admission, neutrophile count and blood glucose at admission were significantly correlated with CRP (p<0.05). Then these factors were further analyzed using multiple regression and only fifth minutes APGAR score, gestational age, birth weight, WBC and neotrophile remained significantly associated with CRP results (Table **3**).

Of total study subjects, 27 (8.9%) neonates died and neonate with low APGAR score (<6) at 1^st^ and 5^th^ minutes were at higher risk of death (OR 5.25,95%CI 1.12-24.80) and (OR 6.04, 95%CI 1.93-18.89, p-0.002), respectively. Neonates who were hypothermic at admission were also more likelihood of death than with febrile counterparts (OR 4.19, 95% CI 1.2-16.21, p-0.038).

## 4 DISCUSSION

Diagnosis of neonatal sepsis is one of the major challenges neonatologists or pediatricians face at NICU because of nonspecific clinical presentation, delayed diagnosis and associated high risk of mortality. CRP has been used as an acute phase reactant to diagnose and follow the course of neonatal sepsis widely studied and available at most centers [1-3].

In this study, CRP was positive in 136 (45%) of neonates with clinical sepsis which was lower than the finding reported by Ramesh which was 69% (9). The sensitivity, specificity, PPV and NPV of CRP were 78.5, 66, 43 and 90.4%, respectively, by taking blood culture as gold standard. Different kinds of literature were reporting a wide range of sensitivity and specificity of 50-90% and 85-95%, respectively. Similarly, the PPV and NPV of CRP vary widely among different study results ranging from 30% to greater 95% [2, 4, 5, 7, 8, 10]. These extreme variations of findings from different kinds of literature could be justified by inclusion criteria, patient clinical condition, time of determination, and technique during the test and the quality of latex reagent.

In our finding, the sensitivity of CRP is high in which such test is recommended to have better sensitivity for developing countries where other better tests are limited and diseases condition tend to undergo aggressive natural course and high case fatality rate [3].

Neonates with both gram positive and gram negative bacterial sepsis were having comparable CRP positivity rate which is in contrast with the report from Tanzania which reported higher CRP positivity rate among neonates with gram-positive bacterial sepsis [13]. Term and normal birth weight neonates with sepsis were more likely to have positive CRP than preterm and LBW newborns which could be justified by the former groups of neonates can mount adequate inflammatory response compared to the later which was also described by Sarah [8] that extremely premature neonates can fail to mount a CRP response. On the other hand, low APGAR score at 5^th^ minutes was strongly associated with positive CRP, our study which was incongruent with the study reported by Hofer [3]. Other perinatal factors like prolonged labor, fetal distress, meconium stained liquor and low first minute APGAR score were not significantly associated with positive CRP and which were also demonstrated in other study. Even though these conditions were stressful for the newborn, most inflammatory conditions go back within 24-36 hours of postnatal age [13].

Abnormal WBC count is one of the earliest signs of systemic inflammatory response syndrome which could be characterized by either leucopenia or leukocytosis. In this study, leukocytosis and neutrophilia were the two most predominant abnormal WBC results and these abnormalities were significantly predicting neonatal sepsis which was also well elaborated by Philip [13-15]. Lower first and fifth APGAR score were strongly associated with both increased WBC and absolute neutrophils count which could be explained by low APGAR score reflects stressful conditions. A similar finding was elaborated in by Geoffrey A *et al* that leukocytosis was seen in asphyxiated neonate [4]. Otherwise, there was no association between WBC count and some clinical factors like birth weight of the neonate, gestational age of the neonate, prolonged labor, Chorioamnionitis, Meconium stained liquor, maternal fever, and the smell of amniotic fluid which was similarly described by Morven S [5]. Our study also showed that high WBC count (>20,000) was significantly associated with culture-proven neonatal sepsis. Similar findings were reported from Tanzania and other similar settings where the sensitivity of increased WBC was significantly associated with higher risk of culture-proven neonatal sepsis [10, 12, 16, 17].

When both WBC count and CRP were combined, their diagnostic accuracy became much better than each test used alone which was also in agreement with the finding reported by other researchers. For example, research finding reported by Flora from Tanzania and Philip from Italy indicated the presence of two abnormal parameters in sepsis screening were yielding sensitivity of 93-100%, specificity of 83%, positive and negative predictive values of 27% and 100%, respectively in detecting culture-proven sepsis [11, 13, 14].

The limitations of the study were the absence of serial CRP determination, qualitative determination of the test, lack of other inflammatory markers like Immature to total neutrophile ratio, procalcitonin and confinement of the study to a single center.

## CONCLUSION

 From this study finding, it can be concluded that CRP alone or in combination with WBC count demonstrated a better screening output in neonatal sepsis evaluation for timely management decision. The higher negative predictive values of these screening tests implied early decision of patients’ management and reduce unnecessary hospital stay and improve rational antibiotic utilization.

## Figures and Tables

**Table 1:** Some demographic and clinical characteristics of neonates with sepsis, at NICU, ATRH May 2016 to April 2017.

**Characteristic**	**Number**	**Percent (%)**
**Age**		
< 7 days	185	61.2
7-28 days	118	38.8
**Gestational age**		
<37 weeks	69	22.9
≥ 37 weeks	234	77.1
**Birth weight**		
<1500gm	18	5.9
1500-2500gm	55	18.2
>2500gm	230	75.9
Number of ANC*		
<4 visit	193	63.8
≥4 visits	110	36.2
**Duration of ROM ¥**		
<18 hours	221	72.8
≥18 hours	82	27.2
**Duration of labor**		
<24 hours	224	73.8
≥24 hours	79	26.2
**Respiratory distress**		
Yes	200	66
No	103	34
**Body temperature**		
Hypothermia	112	37.1
Fever	143	47.3
**Neonatal reflex**		
Intact	106	35.1
Depressed	197	62.9

*ANC: Antenatal Care; **^¥^PROM:** Premature Rupture of Membrane

**Table 2:** Showing the sensitivity, specificity, PPV and NPV of WBC, Neutrophil count and C-reactive protein (CRP), NICU, ATRH, May 2016 to April 2017.

-	Sensitivity	Specificity	PPV	NPV
WBC	59.5%	79.6%	52%	86.1%
Neutrophile	78%	7.1%	24.2%	46%
CRP	78.5%	66%	47.5%	86.4%
WBC+CRP	78.5^%^	83%	60%	93%
CRP in gram positive sepsis	72.2%	92.9%	72.5%	93%
CRP in gram negative sepsis	85.7%	93.6%	73.5%	97%

**Table 3:** Multiple logistic regression showing factors affecting CRP and WBC result, NICU, ATRH, May 2016 to April 2017.

**Variable**	**Positive CRP**			**Leukocytosis**		
-	**OR**	**95%CI**	**P value**	**OR**	**95%CI**	**P-value**
Term	2.48	1.23-4.72	0.006*	1.52	0.991-3.35	0.298
Low first minute APGAR	1.02	0.67-1.71	0.111	1.84	0.97-3.19	0.021*
Low Fifth minute APGAR	2.16	1.17-4.00	0.014*	2.13	1.60-5.15	0.004*
Normal birth weight	2.08	1.01-4.35	0.048*	1.54	0.77-3.07	0.222
Prolonged labor	1.10	0.61-1.97	0.752	1.68	0.91-3.10	0.098
Maternal Chorioamnionitis	1.76	0.68-4.55	0.245	1.35	0.51-5.89	0.544
Meconium stained liquor	1.74	0.878-3.31	0.15	1.44	0.72-2.86	0.306
LONS **Ф**	1.45	0.86-2.43	0.169	1.54	0.76-2.63	0.172
Gram negative bacteria	1.70	0.63-4.59	0.17	4.80	1.45-15.87	0.01*

**Ф** LONS late onset neonatal sepsis, * p- value less than 0.05

## LIST OF ABBREVIATIONS

ATRH: = Asella Teaching and Referral Hospital
CBC: = Complete Blood Count
CSF: = Cerebrospinal Fluid
CRP: = C-Reactive Protein
CPAP: = Continuous Positive Airway Pressure
EONS: = Early Onset Neonatal Sepsis
IAPLONS: = Late Onset Neonatal Sepsis
LP: = Lumber Puncture
NICU: = Neonatal Intensive Care Unit
NPV: = Negative Predictive Value
PPV: = Positive Predictive Value
SVD: = Spontaneous Vaginal Delivery
VLBW: = Very Low Birth Weight
WBC: = White Blood Cell Count
WHO: = World Health Organization
